# Expanding Distribution of Lethal Amphibian Fungus *Batrachochytrium salamandrivorans* in Europe

**DOI:** 10.3201/eid2207.160109

**Published:** 2016-07

**Authors:** Annemarieke Spitzen-van der Sluijs, An Martel, Johan Asselberghs, Emma K. Bales, Wouter Beukema, Molly C. Bletz, Lutz Dalbeck, Edo Goverse, Alexander Kerres, Thierry Kinet, Kai Kirst, Arnaud Laudelout, Luis F. Marin da Fonte, Andreas Nöllert, Dagmar Ohlhoff, Joana Sabino-Pinto, Benedikt R. Schmidt, Jeroen Speybroeck, Frank Spikmans, Sebastian Steinfartz, Michael Veith, Miguel Vences, Norman Wagner, Frank Pasmans, Stefan Lötters

**Affiliations:** Reptile, Amphibian & Fish Conservation Netherlands, Nijmegen, the Netherlands (A. Spitzen-van der Sluijs, E. Goverse, F. Spikmans);; Ghent University, Merelbeke, Belgium (A. Martel, W. Beukema, F. Pasmans);; Natuurpunt Hylawerkgroep Beneden-Nete, Mechelen/Duffel, Belgium (J. Asselberghs);; Technische Universität Braunschweig, Braunschweig, Germany (E.K. Bales, M.C. Bletz, J. Sabino-Pinto, S. Steinfartz, M. Vences);; Biologische Station, Düren, Germany (L. Dalbeck, D. Ohlhoff);; Consultant, Monschau, Germany (A. Kerres);; Natagora, Namur, Belgium (T. Kinet, A. Laudelout);; Biologische Station, Aachen, Germany (K. Kirst); Trier University, Trier, Germany (L.F. Marin da Fonte, M. Veith, N. Wagner, S. Lötters);; Consultant, Jena, Germany (A. Nöllert);; University of Zurich, Zurich, Switzerland (B.R. Schmidt);; Koordinationsstelle für Amphibien und Reptilienschutz in der Schweiz, Neuchâtel, Switzerland (B.R. Schmidt);; Research Institute for Nature and Forest, Brussels, Belgium (J. Speybroeck)

**Keywords:** Batrachochytrium salamandrivorans, Ichthyosaura alpestris, Lissotriton vulgaris, Salamandra salamandra, chytridiomycosis, amphibian, fungi, Belgium, Germany, the Netherlands, Europe, geographic distribution

## Abstract

Emerging fungal diseases can drive amphibian species to local extinction. During 2010–2016, we examined 1,921 urodeles in 3 European countries. Presence of the chytrid fungus *Batrachochytrium salamandrivorans* at new locations and in urodeles of different species expands the known geographic and host range of the fungus and underpins its imminent threat to biodiversity.

Amphibians provide an iconic example of disease-driven global loss in biodiversity. The recently described chytrid fungus *Batrachochytrium salamandrivorans* (*Bsal*) is an emerging pathogen that is driving amphibian populations to local extinction (*1*,*2*). This highly pathogenic fungus causes a lethal skin disease that has so far been restricted to urodele amphibians (newts and salamanders); the fungus was most likely introduced from East Asia into Europe via the pet trade (*2*). In Europe, *Bsal* infection has led to dramatic declines of fire salamander (*Salamandra salamandra*) populations in the Netherlands and Belgium (*2*). Within 7 years after the supposed introduction of the fungus, a population in the Netherlands declined by 99.9% (*3*,*4*). In the United Kingdom and Germany, *Bsal* has been detected in captive salamanders and newts (*5*,*6*). Infection trials suggest that *Bsal* represents an unprecedented threat to diversity of Western Palearctic urodeles (*2*); nevertheless, reports of deaths among salamanders and newts in their naturalized ranges have been restricted to a few populations of a single salamander species in the southern Netherlands and adjacent Belgium (*1*,*3*). Using data from field surveillance, we examined the hosts and the geographic range of *Bsal* in Europe.

## The Study

During 2010–2016, we collected samples of free-living populations of newts and salamanders from 48 sites in the Netherlands, Belgium, and adjacent regions of the Eifel region in Germany (near the border with the Netherlands and Belgium) (Figure; Technical Appendix Table 1). Site selection was based on reported amphibian deaths, apparent negative amphibian population trends, preventive *Bsal* surveillance in susceptible populations, or geographic proximity to known outbreak sites. Samples were also collected at 6 additional sites in Germany and 1 in the Netherlands, which were located >100 km from the nearest known outbreak (Technical Appendix Table 2). Sampling was conducted by swabbing skin (*7*,*8*) of live animals and collecting skin samples from dead animals. All samples were kept frozen at −20°C until they were analyzed for the presence of *Bsal* DNA via real-time PCR, as described (*9*).

**Figure:**
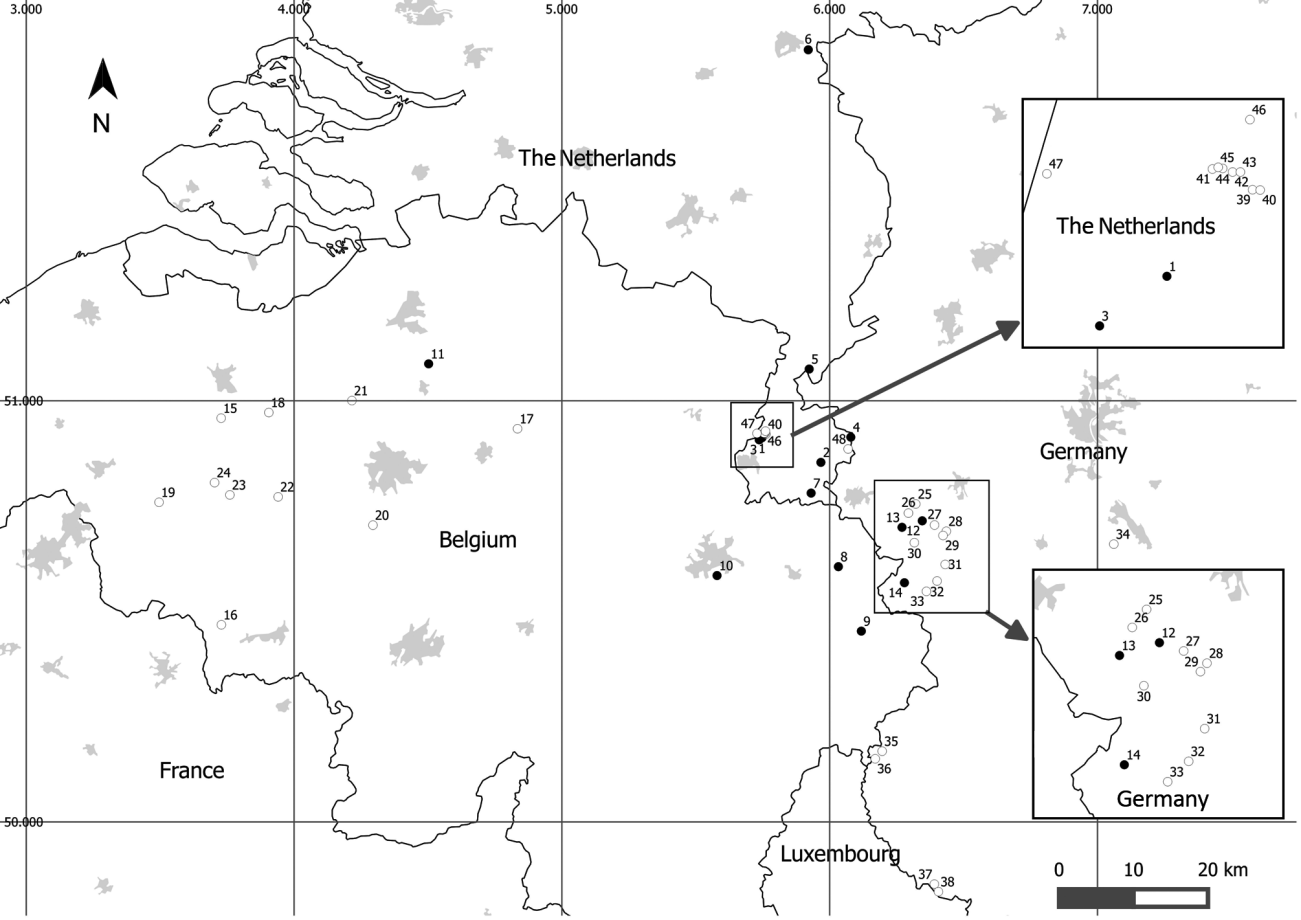
Study sites for collection of amphibians in Western Europe, 2010–2016. Numbers correspond to field sites at which amphibians were collected and examined for *Batrachochytrium salamandrivorans* (*Bsal*) (Technical Appendix). Solid circles, *Bsal* detected; open circles, *Bsal* not detected. Larger cities are indicated in light gray. Note that there are additional sites where the fungus remained undetected (not shown).

Across all 55 sites, we tested 1,019 fire salamanders (43 dead, 976 skin swab samples); at site 14, skin swab samples instead of tissue samples were collected from 16 dead salamanders. We also collected samples from 474 alpine newts (*Ichthyosaura alpestris*; 18 dead, 456 skin swab samples), 239 smooth newts (*Lissotriton vulgaris*; 2 dead, 237 skin swab samples), 80 palmate newts (*Lissotriton helveticus*; all skin swab samples), 79 crested newts (*Triturus cristatus*; all skin swab samples), and 30 Italian crested newts (*Triturus carnifex*; all skin swab samples). To obtain a Bayesian 95% credible interval for prevalence (online Technical Appendix), we used the computational methods of Lötters et al. (*10*). We ran 3 parallel Markov chains with 20,000 iterations each and discarded the first 5,000 iterations as burn-in; chains were not thinned. 

*Bsal* was found at 14 of the 55 sites; infected amphibians were fire salamanders, alpine newts, and smooth newts. Our results demonstrate that the range of *Bsal* distribution may be up to ≈10,000 km^2^ (measured as the surface of a minimum convex polygon encompassing the outermost points) across Germany, Belgium, and the Netherlands (Figure). The presence of *Bsal* in wild alpine newts and smooth newts shows distinct expansion of the known host range in the wild (Technical Appendix Table 1). Furthermore, we document that *Bsal* is present in natural fire salamander populations in Germany (confined to the Eifel region). At some sites, because of our sample sizes, the upper limit of the 95% credible interval for *Bsal* prevalence was as high as 0.7; therefore, we may have failed to detect *Bsal* at these sites (Technical Appendix Table 1). In addition, the fungus may have been present at several sites before first detection. For example, *Bsal* was detected at site 4, where population-monitoring efforts in the years before detection (2000–2013) showed declines in 4 newt species (http://www.ravon.nl/EID_SI_Spitzen_et_al_2016). However, because no samples were collected before 2015, we have no evidence for a causal relationship between the presence of *Bsal* and the declines. We have also recorded the presence of *Bsal* in populations with no evidence of population change so far, such as the incidental findings of dead *Bsal*-positive newts in fyke nets at sites 5 and 11, and the incidental findings of dead *Bsal*-positive fire salamanders at sites 12 and 14. Clinical signs of mycosis, such as lethargy and skin shedding (*1*), were observed at some *Bsal*-positive sites (1, 2, 7, 8, 14) but not at others.

## Conclusions

Our study provides evidence that *Bsal* among wild amphibians in Europe is more widely distributed and affects a wider host range than previously known, which can either indicate recent spread of the fungus or point to historically infected sites that hitherto remained undetected. The presence of *Bsal* in wild populations can easily remain unnoticed because the lesions develop only near the final stage of the disease (*1*). This information is crucial for the design of field surveys for *Bsal* surveillance. Our data might be used to inform a management strategy and to implement the recommendation of the Bern Convention (*11*) to halt the spread of *Bsal* in Europe. Research to search for molecular evidence that the outbreak locations are connected is under way. Chytrid disease dynamics are affected by multiple factors (e.g., temperature regimes [*1*]), and yet undetermined environmental determinants might be essential for disease outbreaks (*12*). Untangling these factors, as well as the modes of *Bsal* spread and its geographic distribution, are points for further research to fully map the problem and identify populations and species at risk.

Technical Appendix Field sites in Europe at which *Batrachochytrium salamandrivorans* fungus was and was not detected in amphibians, 2010–2016.
