# Supplementary material for: Expanding Distribution of Lethal Amphibian Fungus Batrachochytrium salamandrivorans in Europe
Source: Emerg Infect Dis. 2016 Jul;22(7):1286–8. doi: 10.3201/eid2207.160109 (PMC4918153; doi:10.3201/eid2207.160109)
Supplement: Technical Appendix — Field sites in Europe at which Batrachochytrium salamandrivorans fungus was and was not detected in amphibians, 2010–2016. [file 16-0109-Techapp-s1.pdf]

Article DOI: http://dx.doi.org/10.3201/eid2207.160109

Expanding Distribution of Lethal Amphibian Fungus *Batrachochytrium salamandrivorans* in Europe

Technical Appendix

Technical Appendix Table 1. Field sites where *Bsal* was detected, sampled species, numbers of *Bsal*-positive and total sampled specimens*

Site no., location, and amphibian collected	No. *Bsal*-positive/total tested (year)	Observed prevalence (Bayesian 95% credible intervals)	Remarks
The Netherlands			
1, Bunderbos, deciduous forest			
Fire salamander	3/3 (2010)	1.00 (0.42–1.00)	Past mass deaths; 99.9% population decline (1997–2014)
	1/1 (2011)	1.00	
	1/1 (2012)	1.00	
	0/3 (2014)	0 (0–0.61)	
	2/14 (2015)	0.14 (0.04–0.40	
	0/1 (2016)	0	
Alpine newt	1/1 (2013)	1.00	Possibly declining (monitoring started in 2013)†
	1/39 (2014)	0.03 (0.01–0.13)	
	1/10 (2015)	0.10 (0.02–0.43)	
	0/6 (2016)	0 (0–0.43)	
2, Putberg, deciduous forest			
Smooth newt	0/2 (2014)	0 (0–0.70)	Possibly declining†
Alpine newt	0/10 (2014)	0 (0–0.31)	Possibly declining†
	1/1‡ (2014)	1.00	
	1/1‡ (2015)	1.00	
3, Meerssen, garden pond			
Fire salamander	0/1 (2015)	0	No evidence of decline§
Smooth newt	4/43 (2015)	0.09 (0.04–0.21)	No evidence of decline§
Alpine newt	0/9 (2015)	0 (0–0.30)	No evidence of decline§
4, Wormdal, clusters of natural ponds in nature conservation area¶			
Smooth newt	1/22 (2015)	0.05 (0.01–0.21)	87% decline(2000–2013)†#
Alpine newt	0/12 (2015)	0 (0–0.26)	96% decline (2000–2013)†#
5, Pepinusbeekdal, extensive agriculture			
Smooth newt	1/2‡ (2014)	0.50 (0.09–0.88)	No evidence of decline†
6, Berg en Dal, garden pond			
Alpine newt	12/12‡ (2015)	1.00 (0.74–1.00)	Yearly mass deaths; species still present§
7, Vijlenerbosch, deciduous forest			
Alpine newt	0/1 (2013)	0	No evidence of decline§
	0/30 (2014)	0 (0–0.11)	
	1/18 (2015)	0.05 (0.02–0.24)	
Smooth newt	0/8 (2014)	0 (0–0.31)	No evidence of decline§
	0/11 (2015)	0 (0–0.26)	
Palmate newt	0/1 (2014)	0	No evidence of decline§
	0/9 (2015)	0 (0–0.30)	
Belgium			
8, Eupen, deciduous forest			
Fire salamander	1/2 (2013)	0.50 (0.09–0.88)	Deaths, probably fire salamanders severely declining, no monitoring trend available
9, Robertville, deciduous forest			
Fire salamander	16/30 (2014)	0.53 (0.36–0.69)	Deaths, severe decline, monitoring ongoing
10, Liège, deciduous forest			

Site no., location, and amphibian collected	No. *Bsal*-positive/total tested (year)	Observed prevalence (Bayesian 95% credible intervals)	Remarks
Fire salamander	5/5 (2014)	1.00 (0.55–.00)	Deaths
11, Duffel, garden pond			
Alpine newt	2/30‡ (2015)	0.07 (0.02–0.22)	2 dead in fyke; no evidence of decline
Smooth newt	0/16 (2015)	0 (0–0.20)	No deaths; no evidence of decline
Germany			
12, Weisse Wehe, deciduous forest			
Fire salamander	4/11‡ (2015)	0.36 (0.15–0.65)	No evidence of decline†
13, Solchbachtal, mixed forest			
Fire salamander	0/2 (2014)	0 (0–0.70)	Decreased newts and salamanders§
	1/51 (2015)	0.02 (0.01–0.10)	
Palmate newt	0/19(2014)	0 (0–0.18)	Decreased newts and salamanders§
Alpine newt	0/5(2014)	0 (0–0.44)	Decreased newts and salamanders§
14, Belgenbachtal, mixed forest			
Fire salamander	21/22‡ (2015)	0.96 (0.79–0.99)	Remarkable deaths (16 dead), noted only since Nov 2015†

**Bsal, Batrachochytrium salamandrivorans.* Data provide an overview of novel information and previously published data. Site numbers correspond to those on map (Figure).
†Population monitored.
‡Includes individual(s) found dead by chance.
§Anecdotal reports.
¶At this site, crested newts and smooth newts decreased with similar percentages over the same period (−96%; −94%, respectively).
#http://www.ravon.nl/EID_SI_Spitzen_et_al_2016.

Technical Appendix Table 2. Field sites studied where *Bsal* was not detected, number of sampled species and specimens*

Site no., location, and amphibian collected	Number of specimens tested (year)	Observed prevalence (Bayesian 95% credible intervals)	Remarks
Belgium			
15, Nerenbos, deciduous forest			
Fire salamander	30 (2015)	0 (0–0.11)	No evidence of decline†
16, Heilig Geestgoed, deciduous forest			
Fire salamander	30 (2015)	0 (0–0.11)	No evidence of decline†
17, Kasteel van Horst, deciduous forest			
Fire salamander	30 (2015)	0 (0–0.11)	No evidence of decline†
18, Smetledebos, deciduous forest			
Fire salamander	30 (2015)	0 (0–0.11)	No evidence of decline†
19, Kluisbos, deciduous forest			
Fire salamander	30 (2015)	0 (0–0.11)	No evidence of decline†
20, Hallerbos, deciduous forest			
Fire salamander	30 (2015)	0 (0–0.11)	No evidence of decline†
21, Buggenhoutbos, deciduous forest			
Fire salamander	30 (2015)	0 (0–0.11)	No evidence of decline†
22, Raspaillebos, deciduous forest			
Fire salamander	30 (2015)	0 (0–0.11)	No evidence of decline†
23, Haeyesbos, deciduous forest			
Fire salamander	30 (2015)	0 (0–0.11)	No evidence of decline†
24, t Burreken, deciduous forest			
Fire salamander	30 (2015)	0 (0–0.11)	No evidence of decline†
Germany			
25, Lamersiefen, deciduous forest			
Fire salamander	17 (2014)	0 (0–0.19)	No evidence of decline†
	32 (2015)	0 (0–0.11)	
26, Fischbach, deciduous forest			
Fire salamander	36 (2014)	0 (0–0.09)	No evidence of decline; 3 dead-found specimens Tested negative for *Bsal* via histology (2014)†
	51 (2015)	0 (0–0.07)	
Alpine newt	1 (2015)	0	Live-studied specimen by chance; no evidence of decline

Site no., location, and amphibian collected	Number of specimens tested (year)	Observed prevalence (Bayesian 95% credible intervals)	Remarks
Palmate newt	1 (2015)	0	Live-studied specimen by chance; no evidence of decline
27, Kallerbach, deciduous forest			
Fire salamander	24(2015)	0 (0–0.15)	No evidence of decline†
28, Rosbach, deciduous forest			
Fire salamander	47 (2015)	0 (0–0.07)	No evidence of decline†
29, Zweifallshammer, deciduous forest			
Fire salamander	41 (2015)	0 (0–0.08)	No evidence of decline†
30, Peterbach, mixed forest			
Palmate newt	12 (2014)	0 (0–0.26)	No evidence of decline
Alpine newt	4 (2014)	0 (0–0.52)	No evidence of decline
31, Haftenbach, deciduous forest			
Fire salamander	46 (2015)	0 (0–0.08)	No evidence of decline†
32, Sauerbach, deciduous forest			
Fire salamander	22 (2015)	0 (0–0.15)	No evidence of decline†
Alpine newt	1 (2015)	0,00	No evidence of decline
33, Härtgessief, deciduous forest			
Fire salamander	15 (2014)	0 (0–0.19)	Strong evidence of decline†
34, Kottenforst, deciduous forest			
Fire salamander	51 (2015)	(0–0.07)	No evidence of decline
35, Großkampenberg, mixed forest			
Alpine newt	4 (2015)	0 (0–0.52)	No evidence of decline
Palmate newt	1 (2015)	0	No evidence of decline
36, Lützkampen -mixed forest			
Alpine newt	8 (2015)	0 (0–0.31)	No evidence of decline
37, Ferschweiler- mixed forest			
Alpine newt	2 (2015)	0 (0–0.70)	No evidence of decline
Palmate newt	8 (2015)	0 (0–0.31)	No evidence of decline
38, Ernzen, mixed forest			
Fire salamander	4 (2015)	0 (0–0.52)	No evidence of decline†
The Netherlands			
39, Moerveld surroundings (A), Bunderbos vicinity			
Alpine newt	13 (2015)	0 (0–0.22)	No evidence of decline‡
40, Moerveld surroundings (B), Bunderbos vicinity			
Alpine newt	34 (2015)	0 (0–0.11)	No evidence of decline‡
41, Snijdersbergweg 21, garden pond			
Alpine newt	60 (2015)	0 (0–0.06)	No evidence of decline‡
42, Mevr van der Meijstraat 12, garden pond			
Alpine newt	19 (2015)	0 (0–0.18)	No evidence of decline‡
43, Mevr van der Meijstraat 20, garden pond			
Alpine newt	17 (2015)	0 (0–0.19)	No evidence of decline‡
44, Snijdersbergweg 20, 2 garden ponds			
Alpine newt	30 (2015)	0 (0–0.11)	No evidence of decline‡
45, Snijdersbergweg 23b, garden pond			
Alpine newt	15 (2015)	0 (0–0.19)	No evidence of decline‡
46, Broekhoven, garden pond			
Fire salamander	2 (2015)	0 (0–0.70)	No evidence of decline‡
47, Meerssen, deciduous forest			
Fire salamander	57 (2013)	0 (0–0.06)	No deaths; no evidence of decline†
	43 (2014)	0 (0–0.08)	
	29 (2015)	0 (0–0.11)	
	2 (2016)	0 (0–0.70)	
48, Carisberg, deciduous forest			
Alpine newt	8 (2014)	0 (0–0.31)	No information available
Palmate newt	23 (2014)	0 (0–0.14)	No information available
Smooth newt	2 (2014)	0 (0–0.70)	No information available
Additional far-out sites (Germany)			
N.S., Solling, deciduous forest			
Fire salamander	23 (2015)	0 (0–0.14)	No evidence of decline‡
N.S., Ilsenburg, deciduous forest			
Fire salamander	8 (2015)	0 (0–0.31)	No evidence of decline‡
N.S., Lelm, deciduous forest			
Alpine newt	57 (2015)	0 (0–0.06)	No evidence of decline‡
Palmate newt	6 (2015)	0 (0–0.43)	No evidence of decline‡

Site no., location, and amphibian collected	Number of specimens tested (year)	Observed prevalence (Bayesian 95% credible intervals)	Remarks
Smooth newt	16 (2015)	0 (0–0.20)	No evidence of decline‡
Crested newt	29 (2015)	0 (0–0.11)	No evidence of decline‡
N.S., Kleiwiesen, exposed ponds surrounded by deciduous forest			
Alpine newt	27 (2015)	0 (0–0.13)	No evidence of decline‡
Smooth newt	117 (2015)	0 (0–0.03)	No evidence of decline‡
Crested newt	27 (2015)	0 (0–0.13)	No evidence of decline‡
N.S., Waldecker Schlossgrund, deciduous forest			
Fire salamander	22 (2015)	0 (0–0.15)	No evidence of decline‡
N.S., Closewitz, exposed ponds surrounded by deciduous forest			
Crested newt	23 (2015)	0 (0–0.14)	No evidence of decline‡
Additional far-out site (the Netherlands)			
N.S., Veluwe, deciduous forest			
Italian crested newt	0 (2015)	0 (0–0.11)	No evidence of decline‡

**Bsal, Batrachochytrium salamandrivorans*; N.S., not shown on map (Figure). Data provide an overview of novel information and previously published data. Site numbers correspond to those on map (Figure).
†Population monitored.
‡Anecdotal report.
